# Coating Effect on the ^1^H—NMR Relaxation Properties of Iron Oxide Magnetic Nanoparticles

**DOI:** 10.3390/nano10091660

**Published:** 2020-08-24

**Authors:** Francesca Brero, Martina Basini, Matteo Avolio, Francesco Orsini, Paolo Arosio, Claudio Sangregorio, Claudia Innocenti, Andrea Guerrini, Joanna Boucard, Eléna Ishow, Marc Lecouvey, Jérome Fresnais, Lenaic Lartigue, Alessandro Lascialfari

**Affiliations:** 1 Dipartimento di Fisica and INFN, Università degli Studi di Pavia, Via Bassi 6, 27100 Pavia, Italy; matteo.avolio01@universitadipavia.it (M.A.); alessandro.lascialfari@unipv.it (A.L.); 2 Dipartimento di Fisica and INFN, Università degli Studi di Milano, Via Celoria 16, 20133 Milano, Italy; martina.basini@gmail.com (M.B.); francesco.orsini@unimi.it (F.O.); paolo.arosio@unimi.it (P.A.); 3 ICCOM-CNR, via Madonna del Piano 10, 50019 Sesto Fiorentino (FI), Italy; csangregorio@iccom.cnr.it (C.S.); claudia.innocenti@unifi.it (C.I.); 4 Dipartimento di Chimica “U. Schiff” and INSTM, Università degli Studi di Firenze, Via della Lastruccia 3-13, 50019 Sesto Fiorentino (FI), Italy; andrea.guerrini@sns.it; 5 CNRS, CEISAM UMR 6230, Université de Nantes, F-44000 Nantes, France; joanna.boucard@laposte.net (J.B.); elena.ishow@univ-nantes.fr (E.I.); lenaic.lartigue@univ-nantes.fr (L.L.); 6 CSPBAT-UMR CNRS 7244, Université Sorbonne Paris Nord, 74 rue Marcel Cachin, 93017 Bobigny, France; marc.lecouvey@univ-paris13.fr; 7 CNRS, Laboratoire de Physico-chimie des Electrolytes et Nanosystèmes Interfaciaux, Sorbonne Université, PHENIX—UMR 8234, CEDEX 05 F-75252 Paris, France; jerome.fresnais@sorbonne-universite.fr

**Keywords:** magnetic nanoparticles, Superparamagnetism, Nuclear Magnetic Resonance, Magnetic Resonance Imaging, coating, polyelectrolytes

## Abstract

We present a ^1^H Nuclear Magnetic Resonance (NMR) relaxometry experimental investigation of two series of magnetic nanoparticles, constituted of a maghemite core with a mean diameter d_TEM_ = 17 ± 2.5 nm and 8 ± 0.4 nm, respectively, and coated with four different negative polyelectrolytes. A full structural, morpho-dimensional and magnetic characterization was performed by means of Transmission Electron Microscopy, Atomic Force Microscopy and DC magnetometry. The magnetization curves showed that the investigated nanoparticles displayed a different approach to the saturation depending on the coatings, the less steep ones being those of the two samples coated with P(MAA-*stat*-MAPEG), suggesting the possibility of slightly different local magnetic disorders induced by the presence of the various polyelectrolytes on the particles’ surface. For each series, ^1^H NMR relaxivities were found to depend very slightly on the surface coating. We observed a higher transverse nuclear relaxivity, r_2_, at all investigated frequencies (10 kHz ≤ ν_L_ ≤ 60 MHz) for the larger diameter series, and a very different frequency behavior for the longitudinal nuclear relaxivity, r_1_, between the two series. In particular, the first one (d_TEM_ = 17 nm) displayed an anomalous increase of r_1_ toward the lowest frequencies, possibly due to high magnetic anisotropy together with spin disorder effects. The other series (d_TEM_ = 8 nm) displayed a r_1_ vs. ν_L_ behavior that can be described by the Roch’s heuristic model. The fitting procedure provided the distance of the minimum approach and the value of the Néel reversal time (τ ≈ 3.5 ÷ 3.9·10^−9^ s) at room temperature, confirming the superparamagnetic nature of these compounds.

## 1. Introduction

Imaging techniques play a fundamental role in every branch of medicine [1,2,3,4,5,6]. Among these, Magnetic Resonance Imaging (MRI) has played a leading role, as it combines the possibility of obtaining 3D images with a spatial resolution down to a few micrometers, the absence of limits for the penetration depth and the use of non-ionizing electromagnetic radiation [7]. The reconstruction of MRI acquisitions is mainly based on the analysis of the Nuclear Magnetic Resonance (NMR) signal coming from the water protons of different liquids/organs/tissues, on which appropriate magnetic field gradients are applied. The search for a higher sensitivity and the continuous optimization of methods and tools in MRI requires the development of efficient contrast agents (CAs) (i.e., biocompatible and biodegradable materials properly designed in terms of geometry, interactions with water and magnetic properties) that can be injected into the body to produce an optimized image contrast [8,9,10,11,12,13,14]. The presence of CAs, in fact, induces a decrease in the nuclear relaxation times T_1_ and T_2_ of protons, producing a local increase (T_1_-relaxing CAs) or decrease (T_2_-relaxing CAs) of the NMR signal in areas of the body that contain the agent, making them appear with unequal brightness/darkness in the MRI image [15]. Approximately 10 years after the first application on humans of the paramagnetic MRI CAs (Young et al. in 1981 [16]), more complex magnetic nanostructures based on iron oxide particles, typically magnetite Fe_3_O_4_ or maghemite γ-Fe_2_O_3_, were introduced into the market (Endorem^®^/Feridex^®^, Feraheme^®^, Combidex^®^, Clariscan^®^ and Resovist^®^) [17,18,19]. Most of them were withdrawn from the market; nevertheless, Resovist^®^ is still sold in a few countries, and Feraheme^®^ is approved for the treatment of iron deficiency in adult chronic kidney disease patients. However, superparamagnetic properties (which mainly lead to a reduction of the T_2_ of the solvent nuclei), low toxicity and the improved synthesis control on the size, shape and surface of the magnetic nanoparticles (MNPs) (due to recently developed synthesis procedures) make them very versatile from an applicative point of view [20].

The efficiency of such particles for diagnostics has indeed been demonstrated to depend on several magnetic (nature of metal ion, spin topology, magnetic anisotropy), morphological/structural (core diameter, shape, crystallinity degree, coating thickness) and chemical (water exchange dynamics, principally due to coating hydrophilicity, permeability and thickness) parameters [21,22,23,24,25,26]. Moreover, a great potential of MNPs relies on their reactive surface, which can be exploited for the anchorage of several molecules with different functionalities. Indeed, over the last two decades, several research groups have tried to functionalize the nanoparticle surface with specific targeting agents, such as antigens and antibodies, or load them with cargo such as drugs, fluorescent dyes, radiotracers, etc. [27]. Ideally, these nanosystems could selectively reach the targeted tissues and organs, increase the image contrast and, at the same time, release a drug or heat that region (through Magnetic Fluid Hyperthermia [28,29,30,31]) to induce cell death. Thus, these nanoparticles can combine properties useful in diagnostics with other properties compatible with therapy, thus becoming potential *theranostic* agents.

This paper studies the morpho-dimensional, magnetic and relaxometric properties of aqueous dispersions of two series of γ-Fe_2_O_3_ superparamagnetic nanoparticles (with mean diameters d_TEM_ ≈ 17 ± 2.5 nm and ≈ 8 ± 0.4 nm) coated with four different types of biocompatible negative polyelectrolytes. The purpose of our study is to investigate how the different kinds of polymer coatings can influence the behavior of the longitudinal (T_1_) and transverse (T_2_) ^1^H NMR relaxation times of MNP suspensions, which have also been shown to depend on the size of the magnetic core.

## 2. Materials and Methods

### 2.1. Tuning the Coating of Maghemite Nanoparticles

#### 2.1.1. Polyelectrolytes Serving as MNP Coatings

We used four different MNP polyelectrolyte coatings: (i) Poly(acrylic acid), named PAA-A (average M_n_ = 1800 g·mol^−1^), purchased from Sigma–Aldrich (St. Louis, MO, USA) and used as received; (ii) a copolymer issued from the random esterification of poly(methacrylic acid) (PMAA) chains with polyethylene glycol (PEG_2000_), PMAA-*g*-PEG_2000_, named PEG-B (M_n_ = 5.86 × 10^4^ g·mol^−1^); (iii) and (iv), two comb-like polymers fabricated by reversible addition-fragmentation chain transfer (RAFT) based on PMMA and poly(ethylene glycol) methyl ether methacrylate (MAPEG_2000_) with two different chain transfer agents, P(MAA-*stat*-MAPEG_2000_), named respectively PEG-C for the hydrophobic transfer agent (M_n_ = 3.99 × 10^4^ g·mol^−1^) and PEG-D for the hydrophilic transfer agent (M_n_ = 2.87 × 10^4^ g·mol^−1^) [32,33].

#### 2.1.2. Fabrication Procedure of Magnetic Nanoparticles

Low diameter (average magnetic core size d_TEM_ ≈ 8 ± 0.4 nm) maghemite-based MNPs (samples A-8, B-8, C-8 and D-8, with PAA-A, PEG-B, PEG-C and PEG-D coatings, respectively) were synthesized following Massart’s protocol relying on the coprecipitation of iron(II) and iron(III) chloride salts in the presence of ammonium hydroxide [34]. High-diameter (average magnetic core d_TEM_ ≈ 17 ± 2.5 nm) maghemite-based nanoparticles (samples A-17, B-17, C-17 and D-17, with PAA-A, PEG-B, PEG-C and PEG-D coatings, respectively) were prepared by a modified Massart’s method [35]. Briefly, iron chloride salt was dissolved in HCl acidic solutions (2 mol·L^−1^) and deoxygenated. Subsequently, 6.6 mL of FeCl_3_·6H_2_O solution (1 mol·L^−1^) and 1.7 mL of FeCl_2_·4H_2_O solution (2 mol·L^−1^) were mixed together and heated up to 70 °C under an argon atmosphere. Under vigorous stirring, a tetrapropylammonium hydroxide solution (1 mol·L^−1^, 64.4 mL) was injected at a 0.7 mL·min^−1^ rate using a syringe pump and then mixed for an additional 20 min. The two suspensions were oxidized to maghemite by an acidic solution of iron nitrate and redispersed in nitric acid [34]. Purification of the dispersion was performed by successive magnetic decantation steps. A size sorting by selective precipitation was conducted to obtain a narrow polydispersity [36]. Coating with the different polyelectrolytes, chosen for their biocompatibility from the perspective of *in cellulo* MRI, was achieved after a protocol already described in the literature [33]. The polymer powder was added to the targeted acidic dispersion of maghemite MNPs (0.06 wt.%) (for example, for 2.5 mL of iron oxide suspension, 5 mg of PAA-A or 15 mg of PEG-B, C, D were added). A 1.4 mol·L^−1^ solution of ammonium hydroxide was added dropwise under stirring to reach a final pH above 8. Dialysis against Millipore water using Spectra/Por^TM^ membrane (regenerated cellulose) with an 8–10 kDa or 300 kDa cut-off was performed for 48 h to remove the excess of polyelectrolytes, while the solution pH reached around 7 after neutralization.

### 2.2. Characterization Methods

#### Experimental Details

The nanoparticle morphology was investigated by transmission electron microscopy (TEM). Images were recorded using a MO-Jeol 123S0 (80 kV) TEM equipped with a GATAN Orius 11 Megapixel Camera. A few drops of suspensions of the nanosystems were deposited onto holey carbon-coated copper grids (300 mesh) purchased from Agar Oxford Instruments.

Atomic Force Microscopy (AFM), performed by means of a Bruker Nanoscope Multimode IIId AFM system operating in tapping mode in air, was used to estimate the total size of the MNPs (core plus organic coating). The measurements were performed using a silicon rectangular cantilever (NSG01, NT_MDT, length of 120 μm, spring constant of 2.5 N/m and a resonance frequency of about 130 kHz). The samples were prepared by drying a drop of very diluted aqueous solution of MNPs on a mica substrate.

The hydrodynamic diameters of the nanosystems were measured with a Zetasizer Nano ZS ZEN 3600 (Malvern Instruments, Worcestershire, UK). Measurements were collected at 25 °C and averaged over three acquisitions, and correlograms were fitted with a Cumulant algorithm. The results presented are given after a lognormal fitting of the mean size volume histogram.

The electrophoretic mobility of the nanosytems was determined with a Zetasizer Nano ZS ZEN 3600 (Malvern Instruments, Worcestershire, UK). From the electrophoretic mobility, the zeta potential, ζ, was deduced using Smoluchowski’s approximation. All the measurements were performed at 25 °C in disposable folded capillary cells (DTS1070) and repeated three times.

The DC magnetic measurements were carried out by a VSM magnetometer (PPMS Quantum Design Ltd., San Diego, CA, USA) and a SQUID magnetometer (MPMS by Quantum Design Ltd., San Diego, CA, USA) operating in the 2–300 K temperature range and −5 ≤ μ_0_H ≤ +5 Tesla magnetic field range. Zero Field Cooled/Field Cooled magnetizations were acquired in a 5 milliTesla probe magnetic field after cooling the sample without (ZFC) and with (FC) the applied field. Due to the small quantity of synthetized products, the magnetic material content in the samples could not be estimated with accuracy. This inaccuracy and the large experimental error in the sample weight only allowed us to assume a rough estimate of the saturation magnetization.

The NMR-dispersion profiles were collected at room temperature by measuring the T_1_ and the T_2_ relaxation times, varying the Larmor frequency of the investigated nuclei (2πν_L_ = γB_0_, where γ = 2.67513 × 10^8^ rad s^−1^ T^−1^ is the gyromagnetic factor of ^1^H, from 10 kHz up to 60 MHz). For low-frequency relaxation measurements (from 0.01 MHz to 7.2 MHz), the Fast-Field-Cycling technique was used by means of a Smartracer Stelar NMR relaxometer. High-frequency relaxation measurements (up to 60 MHz) were performed using a Stelar Spinmaster Fourier transform nuclear magnetic resonance spectrometer. For ν_L_ < 7.2 MHz, pre-polarized Saturation Recovery (for T_1_) and spin-echo (for T_2_) sequences were adopted. For frequencies ν_L_ > 7.2 MHz, non-pre-polarized Saturation Recovery (SR) and Carr Purcell Meiboom Gill (CPMG) pulse sequences were used for the T_1_ and T_2_ measurements, respectively.

## 3. Results and Discussion

### 3.1. Nanoparticles Synthesis

**Figure 1:**
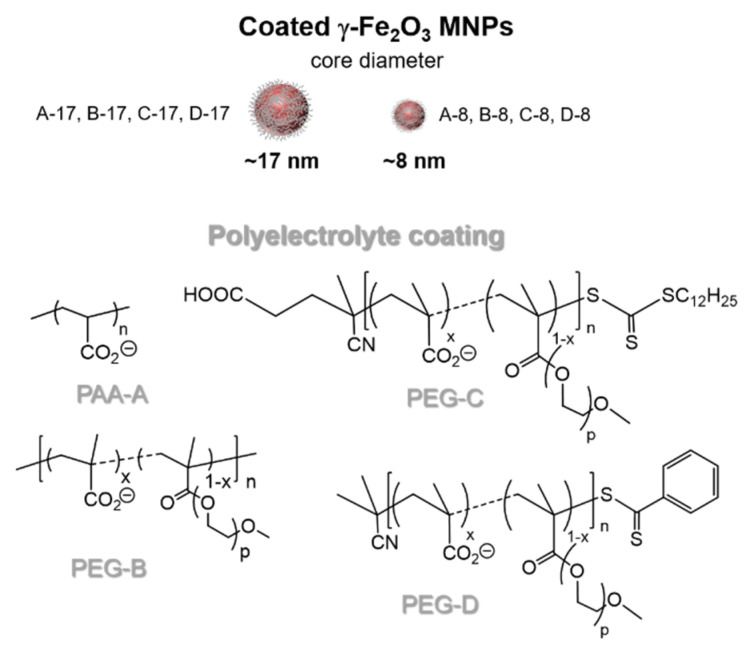
Structures of the investigated MNPs as a function of their core diameter and polyelectrolyte coating, depicted as PAA-A, PEG-B, PEG-C and PEG-D.

The coatings of maghemite MNPs were chosen so that they met three main objectives: easy synthetic access, biocompatibility and hydrophilicity. The structures of the four used polyelectrolytes (PAA-A, PEG-B, PEG-C and PEG-D) are sketched in Figure 1. To confer stealth properties to the coated NPs, PEGylated chains, well known to help nanoparticles evade the mononuclear phagocytic system after in vivo injection, were added to the polymer backbone by using post-esterification (PEG-B) or reversible addition-fragmentation chain transfer polymerization (PEG-C and PEG-D). Two series of coated MNPs, differing by the size of the inorganic core, were actually generated by adding an excess of each polyelectrolyte to an acidic solution of maghemite nanoparticles. After dialysis against Millipore water, alkalinization using ammonium hydroxide was performed so as to favor the anchoring of the polyelectrolyte carboxylate units to the naked surface of the iron oxide nanoparticles.

### 3.2. Morphological Characterization

**Figure 2:**
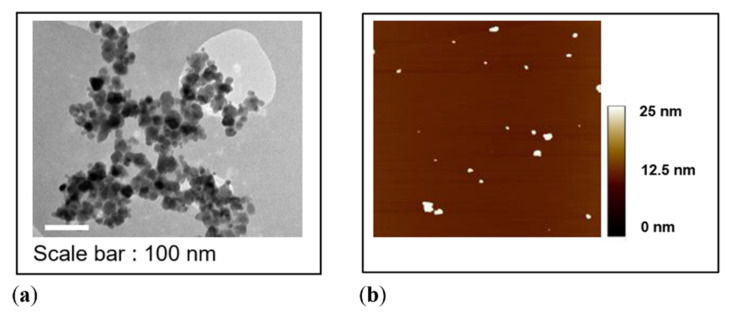
Representative images of sample A-17 obtained by means of: (**a**) bright field TEM and (**b**) AFM over an area of 3 × 3 µm^2^.

**Figure 3:**
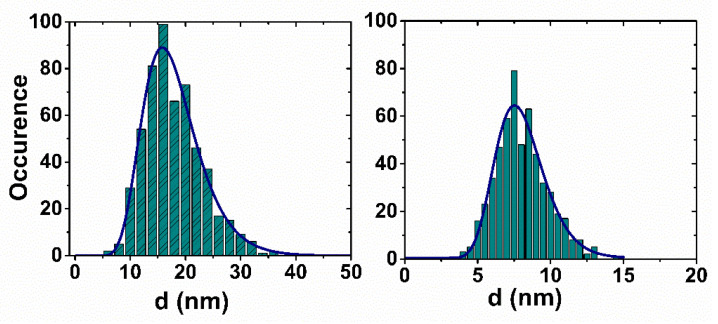
Histograms reporting the distribution of the core sizes for the two series of MNPs, as obtained from TEM analysis (A-17, left and A-8, right). The distributions are fitted to a log-normal function; the mean value and the standard deviation are reported in Table 1.

Both series of samples consist of spherical MNPs, as deduced from the TEM and AFM images, which are presented in Figure 2a,b for A-17. Representative histograms of the core size for A-17 (first series) and A-8 (second series) are reported in Figure 3, while the average and standard deviation of the core diameter distribution are reported in Table 1. After statistical counting of more than 300 nanoparticles, performed using the ImageJ software, the average diameter and the standard deviation were determined by fitting the data to a log-normal distribution:

(1) $$p_{x}{(x,\mu }_{y}{,\sigma }_{y})\;=\;\frac{1}{\sqrt{2\pi }\sigma_{y}x}\exp\left[-\frac{1}{2}{\left(\frac{\mathrm{lnx}\;-{\;\mu }_{y}}{\sigma_{y}}\right)}^{2}\right]$$

where x represents the different values of the diameter, μ_y_ = ln(d_TEM_), where d_TEM_ is the mean diameter, and σ_y_ is the standard deviation. The size distribution for each sample is within 16% around the mean value for both series (Table 1), i.e., large enough to include the mean values of the others samples of the same series.

For the samples of the first series (high diameter), the MNPs’ morphology was also investigated by Tapping Mode Atomic Force Microscopy, which allowed for the evaluation of the overall size of the MNPs, i.e., the diameter of the magnetic core together with its coating. Besides, AFM can distinguish the presence of MNP agglomerates and single MNPs, as shown in the topographic image of Figure 2b. As expected, the MNPs’ average diameter d_AFM_ obtained by AFM is greater than the diameter estimated from the TEM data, due to the presence of the polymeric coating, whose thickness, as calculated from the difference of the diameters obtained through the two different techniques [(d_AFM_ − d_TEM_)/2 (data not reported)], is in the order of 1 ÷ 1.5 nm, depending on the sample.

All of the samples have a negative zeta potential due to the presence of acrylate units on the various polyelectrolytes. Each sample has a zeta potential in the −28 to −48 mV range, indicating a good colloidal stability. The hydrodynamic diameters of the magnetic nanoparticles with a core of 17 nm vary little with the nature of the stabilizing polyelectrolyte and remain within a range of 71 to 85 nm. The increase in diameter, when compared with that obtained by TEM, is consistent with the presence of the polyelectrolyte and a solvation layer on the surface of the nanoparticles. Likewise, the value of 21 nm for the hydrodynamic diameter of the 8-nm nanoparticles samples stabilized by the PAA is coherent with the presence of the polyelectrolyte and of the solvation layer.

### 3.3. Magnetic Measurements

**Figure 4:**
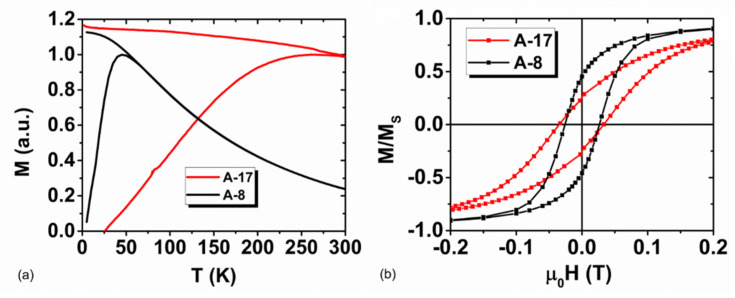
(**a**) ZFC/FC magnetization curves collected with a magnetic field μ_0_H = 5 × 10^−3^ Tesla and (**b**) low field hysteresis loops at 2.5 K for A-17 and A-8.

The ZFC/FC magnetization curves are reported in Figure 4a for the samples A-17 and A-8, which were measured in the form of powders. The temperature of the maximum in the ZFC curve, commonly identified as the blocking temperature of the system, occurs at T_B_ ~45 K for the smaller diameter MNPs. For the larger diameter MNPs, the maximum is broadened around the end of the measuring temperature range (T_B_ ≥ 260–300 K), suggesting that this latter series of samples is in a sort of transition between “blocked/unblocked” (superparamagnetic) regimes at room temperature. As a reminder, T_B_ is proportional to the competition between the magnetic energy barrier (Ea ≈ K_eff_V) and the magnetization reversal process, which, in turn, increases with the effective anisotropy constant (K_eff_) and the volume (V) of the MNPs. Thus, the large difference observed in the T_B_ values of the two series reflects this dependence.

**Figure 5:**
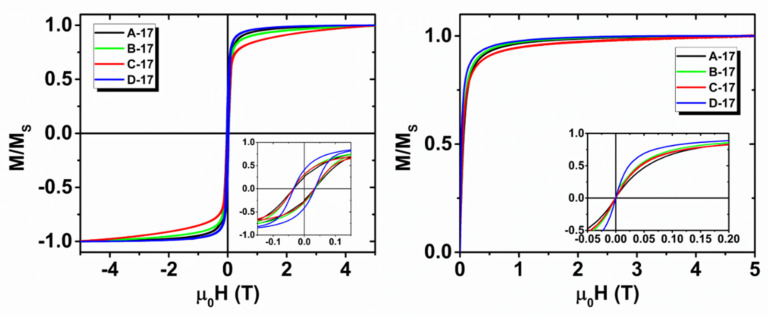
Magnetization curves at 2.5 K (left panel) and 300 K (right panel) for the first series. In the insets, details of the curves at low magnetic fields are shown.

The magnetization curves acquired at low (2.5 K) and high (300 K) temperatures are shown in Figure 5 for the 17-nm series. The curves are normalized to the corresponding saturation magnetization (M_s_) value to better compare their shape features. Samples of the 17-nm series present a similar coercivity (µ_0_H_C_ = 35 milliTesla) at a low temperature, a similar magnetic remanence, M_R_/M_s_ = 0.3 at 2.5 K, and a similar susceptibility, χ, at 300 K, with the exception of D-17, which displays slightly higher M_R_ and χ values. On the contrary, a different approach to saturation (high field region), particularly evident at a low temperature, is observed among the samples; these can be ordered, from slowest to fastest to reach saturation, in the following sequence: C-17, B-17, A-17 and D-17.

A linear approach to saturation is commonly reported in nanoparticle systems and is related to the spin disorder on the particle surface, which affects the magnetization alignment upon increasing the field. In the present case, the different approach that was observed could thus be ascribed to the different modifications of the particle surface induced by: (i) slight variations in the synthesis procedure and/or (ii) the presence of different coatings. Due to their interconnection, distinguishing between these two contributions is not an easy task and will require a more detailed analysis, which is beyond the scope of this work. Interestingly, we can note that a negligible coercivity is recorded in the magnetization curves at room temperature (300 K) for all the samples, suggesting that the transition to the “unblocked” state mentioned above occurred for most of the particles of the 17 nm series at this temperature. The magnetization curves for the second series (not shown) present roughly similar features among the samples at low fields, with µ_0_H_C_ = 25 milliTesla and M_R_/M_s_ = 0.4 at 2.5 K. A comparison of the low field hysteresis at 2.5 K for representative samples of the two series is shown in Figure 4b.

### 3.4. ^1^H NMR Relaxation

Proton NMR relaxation in superparamagnetic colloids occurs because of the fluctuations of the dipolar magnetic coupling between nanoparticle magnetization and proton spins. The relaxation rate is described by an outer sphere model that includes the Curie relaxation, where the dipolar interaction fluctuates because of both the translational diffusion process and the Néel reversal (i.e., the flip of the magnetization vector from one direction of the easy magnetization axis to the opposite one). The sensitivity of MNPs as contrast agents was evaluated through the nuclear relaxivities r_1_ (longitudinal relaxivity) and r_2_ (transverse relaxivity), which were calculated by means of the following equation:

r_i_ = [(1/T_i_)_meas_ − (1/T_i_)_dia_ ]/C  i = 1, 2 (2)

where (1/T_i_)_meas_ is the value measured on the samples, (1/T_i_)_dia_ is the relaxation rate of the dispersant in the absence of superparamagnetic nanoparticles and C is the iron molar concentration within the sample.

#### 3.4.1. Experimental Data

##### 17 nm MNPs (1st Series)

**Figure 6:**
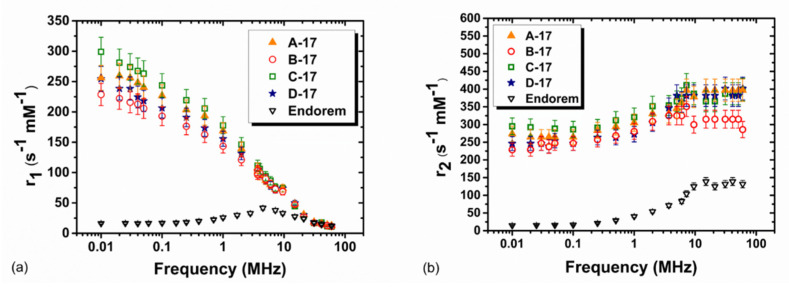
(**a**) Longitudinal r_1_ and (**b**) transverse r_2_ NMRD profiles collected at room temperature in the Larmor frequency range $0.01\leq \nu_{L}\leq 60\;\mathrm{MHz}$ for the first series of polymer-coated MNPs. For comparison, the relaxivity values of Endorem, as reported by Basini et al., are shown [22].

The experimental longitudinal relaxivity profile (r_1_) of the 17-nm series of MNPs is represented in Figure 6a. All samples show a continuous increase of the longitudinal relaxivity lowering the Larmor frequencies, with no detectable maximum. This behavior can be explained qualitatively by taking into account the energy related to the crystal’s internal magnetic anisotropy at a low frequency. The absence of a maximum is common for spherical maghemite-based particles with diameters d_TEM_ above approximately 15 nm. However, here the flattening of the r_1_(ν) curves at a low frequency, which is expected for high anisotropy systems, is not observed [37].

The transverse relaxivity vs. frequency behavior (Figure 6b) is similar for all samples, and at a high magnetic field μ_0_H ~1.41 Tesla (close to the clinical one), r_2_ reaches the value ~285 mM^−1^s^−1^ for sample B-17 and ~400 mM^−1^s^−1^ for samples A-17, C-17 and D-17. At Larmor frequencies ν_L_ > 5–10 MHz, sample B-17 shows r_2_ values smaller than those of the other samples, also slightly reflecting differences in r_1_. The spin disorder, induced by the different polymer and/or the agglomeration effects, possibly generate a lower magnetization value in the case of sample B-17. Table 2 summarizes the r_1_, r_2_ and r_2_/r_1_ values at the two frequencies, namely 60 MHz and 15 MHz. The values are compared to those of Endorem, a commercial T_2_ contrast agent, no longer used since 2012, but which still remains a good reference for assessing the relaxation efficiency of relaxing T_2_ superparamagnetic nanoparticles. The r_2_/r_1_ value is greater than 2, indicating that all the ferrofluids act as negative contrast agents, this value being the threshold conventionally used to distinguish T_1_-relaxing agents and T_2_-relaxing agents [38].

##### 8 nm MNPs (2nd Series)

**Figure 7:**
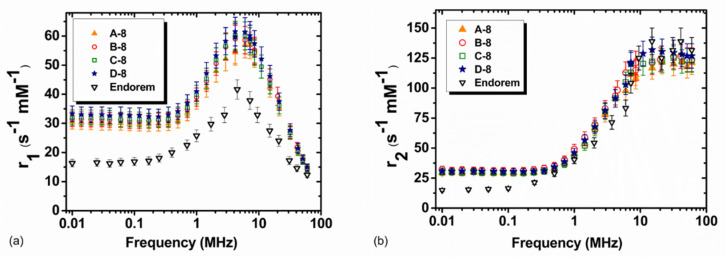
(**a**) Longitudinal r_1_ and (**b**) transverse r_2_ NMRD profiles collected at room temperature in the Larmor frequency range $0.01\leq \nu_{L}\leq 60\;\mathrm{MHz}$ for the second series of polymer-coated MNPs. For comparison, the relaxivity values of Endorem, as reported by Basini et al., are shown [22].

The relaxivity profiles of the 8-nm series of MNPs are presented in Figure 7. From Figure 7a, it is possible to observer that the longitudinal relaxivity r_1_ of this series of MNPs behaves as expected for ultrasmall superparamagnetic particles. The maximum of the longitudinal relaxivity is located between 1 and 20 MHz, and diminishes just by a factor of ~4 at 60 MHz. For sample A-8, the maximum is shifted towards a higher frequency, suggesting slightly smaller sizes. At low fields, all samples present a slight dispersion at 300–400 kHz, indicating that, at room temperature, their magnetization is not completely locked along the magnetic ‘easy’ axis.

In Table 3, the relaxivities of γ-Fe_2_O_3_ nanoparticles are compared to those of Endorem.

The transverse relaxivity vs. frequency behavior (Figure 7b) is similar for all samples, and at a high magnetic field μ_0_H ~1.41 Tesla, r_2_ reaches the value ~125 mM^−1^s^−1^. Then, the transverse relaxometric performance of these samples at the typical clinical frequency ~60 MHz (crucial for darkening the MRI images and thus increasing the sensitivity) is comparable to that of the commercial CA. Additionally, one can note that the frequency behavior of the r_2_ relaxation curve is also similar to that of the commercial compound, although the latter shows lower values for ν < 7 MHz.

It is interesting to note that the r_2_/r_1_ ratio at 60 MHz assumes a value of ~8 for our MNPs and ~11 for Endorem^®^. The r_2_/r_1_ values at 60 and 15.1 MHz are comparable to those of Endorem, indicating that the two substances have a very similar efficiency as T_2_ contrast agents.

#### 3.4.2. Analysis of NMR Results

To analyze the NMR longitudinal relaxivity profiles (i.e., r_1_ vs. frequency) at room temperature, the heuristic model of Roch et al. [37] was employed. We were able to use this model (valid for an ensemble of single nanoparticles) because the coincidence of r_1_ and r_2_ values for each sample at low frequencies, approximately ν < 0.1 MHz, ensured the absence of particle aggregation at the dilution used for the NMR measurements. In more detail, the longitudinal NMRD profiles were fitted using the following expression:

(3) $$\frac{1}{T_{1}}\;=\;\frac{32\pi }{135,000}\mu_{\mathrm{SP}}^{2}{\;\gamma }_{I}^{2}\;\frac{N_{A}\;C}{\mathrm{RD}}7P\frac{L\left(x\right)}{x}J^{F}{\;[\Omega (\omega }_{S}{,\omega }_{0}{),\tau }_{D}{,\tau }_{N}\;]\;+\;[7Q\frac{L\left(x\right)}{x}\;+\;3\left(1\;-{\;L}^{2}\;\left(x\right)\;-\;2\frac{L\left(x\right)}{x}\right)]\;\cdot {\;J}^{F}{\;(\omega }_{L}{,\tau }_{D}{,\tau }_{N}{)\;+\;3L}^{2}\left(x\right)\cdot J^{A}\left(\sqrt{{2\omega }_{I}{\;\tau }_{D}}\right)$$

where $\mu_{SP}$ is the effective magnetic moment of the MNPs experienced by the ^1^H nuclei, $\gamma_{I}$ is the proton gyromagnetic ratio, $N_{A}$ is the Avogadro’s number, *C* is the molar concentration of iron in the MNPs, *R* is the minimum approach distance between the protons and MNPs, *L*(*x*) is the Langevin’s function (*L*(*x*) = cothx-1, where $x=\frac{\mu_{SP}B_{0}}{k_{B}T}$), *D* is the diffusion coefficient of the medium, $\tau_{D}\;$= *R*^2^/*D* is the diffusion time that characterizes the fluctuation of the hyperfine interaction between the nuclear magnetic moments of the ^1^H nuclei of the solvent (here water) and the nanoparticle magnetic moment, $\tau_{N}=\tau_{0}e^{\frac{KV}{k_{B}T}}$ is the Néel relaxation time at room temperature, and $\omega_{S}$ and $\omega_{I}$ are the electron and proton transition frequencies, respectively. The parameters *P* and *Q* are related to the degree of magnetic anisotropy of the system, being the weight of the spectral density functions *J^A^* (Ayant, high fields) and *J^F^* (Freed, low fields), respectively. In particular, *P* = 0 and *Q* = 1 for highly anisotropic systems, while *P* = 1 and *Q* = 0 for weakly anisotropic systems.

For the 17-nm samples, we were not able to fit the experimental data because their size was at the limit of validity of the Roch’s heuristic model (for which nanoparticles should have a mean diameter < 20 nm). Moreover, the fitting process was made difficult by the broad size distribution for all samples (see Figure 3). The non-applicability of the Roch’s model to this series of MNPs was confirmed by the fact that it did not predict any r_1_ increase at the lowest frequencies, as displayed by our experimental data.

**Figure 8:**
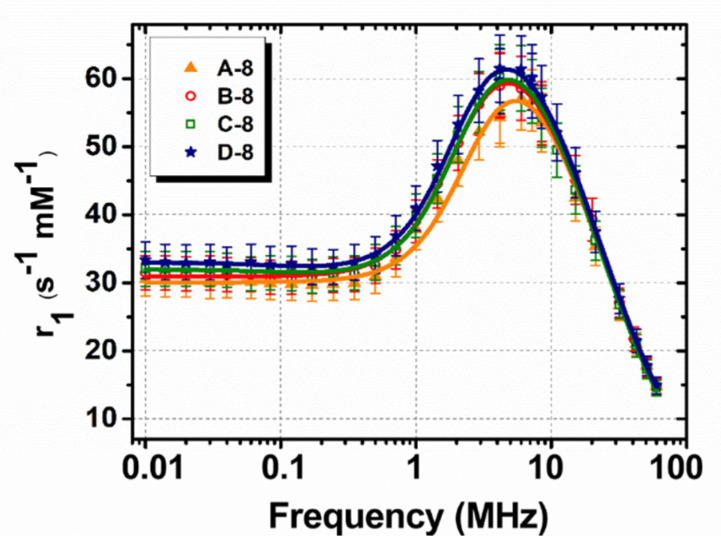
Longitudinal r_1_ NMRD profiles (symbols) collected at room temperature in the Larmor frequency range $0.01\leq \nu_{L}\leq 60\;\mathrm{MHz}$ for the 8-nm series of polymer-coated MNPs. The solid lines represent the best fit obtained by applying the Roch’s model (see text).

Conversely, for the smaller MNPs (second series of samples), we were able to fit the experimental r_1_ data. In Figure 8, the r_1_ fitting curves obtained by means of the Roch model for samples A-8, B-8, C-8 and D-8 are shown. The parameters of physical interest, obtained by the fit of the experimental data of Figure 8 to Equation (3) (deduced from the Roch’s model), are the saturation magnetization M_s_, the magnetic core radius r, the distance R of minimum approach (of the bulk water protons to the MNP magnetic center) and the Néel relaxation time τ_N_.

In the fitting procedure, we let the saturation magnetization M_S_ parameter vary between 60 and 70 Am^2^/kg_γ-Fe2O3,_ as expected from the literature data for particles with a similar size and substantially confirmed by our magnetic measurements for our samples. For the particle radius, by considering the size distribution width, we fixed an upper limit of r ≈ 5 nm. For the water diffusion coefficient, we used the theoretical value D = $2.3\;\times \;10^{-9}{\;m}^{2}s^{-1}$ at 293 K.

For all samples, we observed the following: (i) The saturation values, constrained in the range specified above, allowed for the fitting procedure convergence, obtaining M_s_ = 70 ± 4 Am^2^/kg_γ-Fe2O3_; (ii) The core radius r was slightly higher than the one estimated by TEM (r = 5.0 ± 0.4 nm), a result possibly consistent with the width of the size distributions (Figure 3); (iii) The distance of the minimum approach was 1 ÷ 2 nm greater than the core radius. This latter result points out the tendency of the coating to prevent the diffusion of water inside itself; (iv) The values of τ_N_ were consistent with the ones typical for nanoparticles of this size, in particular τ_N_ ≈ 3.5 ÷ 3.9 × 10^−9^ s.

## 4. Conclusions

In this work, we employed NMR relaxometry to investigate the dependence of the MRI contrast efficiency (i.e., the nuclear relaxivities) on the organic coating of maghemite-based MNPs. In particular, we studied MNPs dispersed in water with two different diameters (d_TEM_ ≈ 8 ± 0.4 nm and ≈ 17 ± 2.5 nm) and four different coatings, i.e., PAA, PMAA-g-PEG and two P(MAA-*stat*-MAPEG) with different transfer agents. A structural, morpho-dimensional and magnetic characterization of the nanoparticles was performed by means of Transmission Electron Microscopy (TEM), Atomic Force Microscopy (AFM) and DC magnetometry. The magnetization curves, particularly at a low temperature, displayed a different approach to saturation depending on the coating, the M_s_ approaching rate of the sample coated with hydrophobic P(MAA-*stat*-MAPEG) being the slowest one, followed by that coated with PMAA-g-PEG. These results seem to suggest that the hydrophobic P(MAA-*stat*-MAPEG) and PMAA-g-PEG coatings favor a higher spin disorder at the particle surface. The r_1-_NMRD profiles show the same behavior for samples with the same core size but with different coatings, indicating that the type of coating used in this work does not evidently influence the longitudinal relaxometric properties. For the transverse relaxivity, we observed a similar trend, except for the sample of the 17-nm series coated with PMAA-g-PEG, which had a lower r_2_, in particular for ν_L_ > 5–10 MHz. Remarkably, all samples showed high r_2_ values at 60 MHz (~120 mM^−1^s^−1^ for d_TEM_ ≈ 8 nm and 300–400 mM^−1^s^−1^ for d_TEM_ ≈ 17 nm), which were comparable to or higher than the transverse relaxivity of the commercial compound Endorem^®^. Thus, our samples are promising superparamagnetic T_2_ contrast agents for MRI, especially in the case of d_TEM_ ≈ 17 nm. This conclusion is supported by the values of the r_2_/r_1_ ratio, which generally provides an indication as to how magnetic nanoparticles may behave in their application as contrast-enhancing agents. In our case, for the larger diameter series and at the most used clinical frequency (ν_L_~60 MHz), the r_2_/r_1_ values were three times larger than the one for Endorem, allowing us to envision a possible superparamagnetic CA dose reduction in clinical use.

## Figures and Tables

**Table 1:** Mean diameters ± standard deviation of the inorganic cores for the first and second series, obtained by TEM.

Sample	d_TEM_ nm
first series samples	17.0 ± 2.5
second series samples	8.0 ± 0.4

**Table 2:** Longitudinal and transverse relaxivity values and their ratio at 15 and 60 MHz, for the 1st series of MNPs aqueous dispersions at room temperature.

Sample	Frequency	r_1_ (s^−1^mM^−1^)	r_2_ (s^−1^mM^−1^)	r_2/_r_1_
A-17	60 MHz	12.4 (1.0)	396.8 (31.7)	32
	15 MHz	48.4 (3.9)	396.8 (31.7)	8.2
B-17	60 MHz	11.3 (0.9)	285.7 (22.8)	25.3
	15 MHz	47.1 (3.8)	314.9 (25.2)	6.7
C-17	60 MHz	10.9 (0.9)	398.7 (31.9)	36.6
	15 MHz	44.5 (3.6)	365.5 (29.2)	8.2
D-17	60 MHz	12.6 (1.0)	401.8 (32.1)	31.9
	15 MHz	49.9 (4.0)	381.7 (30.5)	7.6
Endorem	60 MHz	12.3 (1.0)	131.6 (10.5)	10.7
	15 MHz	27.5 (2.2)	138.9 (11.1)	5

**Table 3:** Longitudinal and transverse relaxivity values and their ratio at 15 and 60 MHz, for the 2nd series of MNPs aqueous dispersions at room temperature.

Sample	Frequency	r_1_ (s^−1^mM^−1^)	r_2_ (s^−1^mM^−1^)	r_2/_r_1_
A-8	60 MHz	14.7 (1.2)	121.0 (9.7)	8.2
	15.1 MHz	42.5 (3.4)	116.7 (9.3)	2.7
B-8	60 MHz	14.7 (1.2)	123.7 (9.9)	8.4
	15.1 MHz	45.1 (3.6)	121.1 (9.7)	2.7
C-8	60 MHz	14.5 (1.2)	123.1 (9.9)	8.5
	15.1 MHz	43.6 (3.5)	122.13 (9.8)	2.8
D-8	60 MHz	14.9 (1.2)	126.7 (10.1)	8.5
	15.1 MHz	46.2 (3.7)	131.9 (10.6)	2.9
Endorem	60 MHz	12.3 (1.0)	131.6 (10.5)	10.7
	15 MHz	27.5 (2.2)	138.9 (11.1)	5
